# Among the DLTs: Holochain for the Security of IoT Distributed Networks—A Review and Conceptual Framework

**DOI:** 10.3390/s25133864

**Published:** 2025-06-21

**Authors:** Shereen Ismail, Raouf Mehannaoui, Eden Teshome Hunde, Hassan Reza

**Affiliations:** 1 Merit Network Inc., University of Michigan, Ann Arbor, MI 48108, USA; 2 Automatic and Production Laboratory, Department of Industrial Engineering, Batna 2 University, Batna 05000, Algeria; raouf.mehannaoui@univ-batna2.dz; 3 Department of Electronics and Informatics, Vrije Universiteit Brussel, Pleinlaan 2, 1050 Brussels, Belgium; eden.teshome.hunde@vub.be; 4 Department of Electrical and Computer Engineering, Jimma Institute of Technology, Jimma University, Jimma 378, Ethiopia; 5 School of Electrical Engineering and Computer Science, University of North Dakota, Grand Forks, ND 58202, USA; hassan.reza@und.edu

**Keywords:** Holochain, IoT networks, security, DLTs, blockchain, decentralization

## Abstract

IoT devices are typically resource-constrained, with limited computational power, storage, and energy. Holochain, an emerging distributed ledger technology (DLT), offers the benefits of blockchain while overcoming its limitations, such as the reliance on consensus algorithms and a globally synchronized ledger. As a result, Holochain has garnered attention in the research community as a promising solution for distributed IoT applications. This paper reviews various DLTs in IoT distributed networks, focusing on the motivation for utilizing Holochain in these environments. We explore its key applications, challenges, and research insights. We propose the *HoloSec* framework, a conceptual security framework for IoT distributed networks that leverages Holochain’s agent-centric architecture, advanced cryptography, and machine learning (ML). The paper also illustrates the setup and implementation of a Holochain-based IoT network for a healthcare scenario and compares the performance of Holochain with traditional blockchain solutions. Initial experimental results show that Holochain achieves a latency of around 50 ms for data publishing and 30 ms for retrieval, with a throughput of approximately 20 transactions per second (TPS) on a single node, significantly outperforming blockchain, which shows higher latency (200 ms publish, 100 ms retrieve) and lower throughput (10 TPS). Finally, we examine key challenges associated with Holochain and outline future research directions aimed at enhancing its interoperability, scalability, security, and regulatory compliance in IoT environments.

## 1. Introduction

Despite their potential to transform digital ecosystems, distributed ledger technologies are not immune to critical threats that challenge their security, scalability, and privacy guarantees. Traditional distributed ledger technologies (DLTs) like blockchain face issues such as Sybil attacks, 51% attacks, double-spending, data immutability abuse, privacy leakage, and scalability limitations, especially when deployed in dynamic and resource-constrained environments like the IoT [1,2,3]. These threats undermine trust, delay adoption, and complicate regulatory compliance, highlighting the need for more resilient, lightweight, and adaptive Distributed Ledger Technology (DLT) architectures.

In recent years, the combined use of blockchain and machine learning has emerged as a promising approach to strengthening IoT security [4,5,6]. DLT-based systems offer decentralized identity management and tamper-evident audit trails, while ML enables real-time anomaly detection, intrusion detection, and intelligent threat response. Together, they provide complementary strengths for securing resource-constrained IoT environments. Prior studies have applied these technologies in sectors such as healthcare, supply chains, and wireless sensor networks, showing improvements in trust, transparency, and attack resilience. However, limitations remain regarding their scalability, latency, and energy efficiency—motivating the exploration of next-generation DLT architectures, such as Holochain.

Holochain represents the beginning of a new era for open-source decentralized DLTs that can integrate with ML, offering a transformative alternative to traditional blockchain systems. Unlike blockchain, which relies on bulky data storage and centralized consensus mechanisms, Holochain is designed to operate without the need for global synchronization or resource-intensive data storage, making it a more efficient and scalable solution for decentralized applications [7]. By utilizing an agent-centric model and Distributed Hash Tables (DHTs), Holochain allows for seamless P2P communication and data exchange without the overhead typically associated with blockchain technologies.

**Figure 1:**
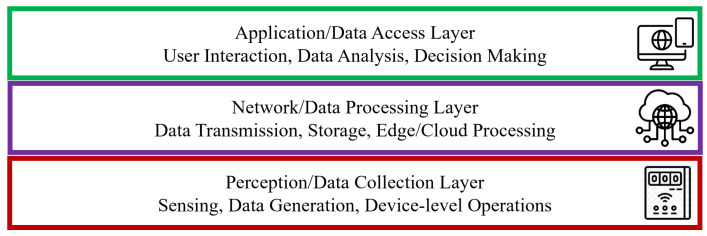
Standard IoT layered architecture with three main layers.

Figure 1 illustrates the three-layered architecture of IoT systems, consisting of the Perception/Data Collection Layer, Network/Data Processing Layer, and Application/Data Access Layer. This layered model provides a clear framework for understanding the flow of data in decentralized IoT environments, from device-level sensing and data generation to edge/cloud processing and ultimately user-facing applications involving analysis and decision-making. As the IoT continues to grow and proliferate across various industries, the need for decentralized, secure, and scalable solutions to manage and share IoT data has become increasingly critical.

Each layer of the IoT architecture is susceptible to specific security threats and vulnerabilities that adversaries can exploit. At the Perception Layer, attacks such as data spoofing, clone node injection, and unauthorized device access can corrupt sensor readings and disrupt data integrity. Solutions in the literature include lightweight cryptographic schemes, secure boot mechanisms, and tamper-proof hardware modules. DLTs can mitigate these risks through decentralized identity management and immutable device logs. By assigning cryptographically verifiable identities to IoT devices and maintaining tamper-evident audit trails, DLTs ensure that only legitimate devices can participate in the network and that all interactions are transparently recorded. This enhances device trustworthiness and supports secure data provenance from the moment of collection.

The Network Layer faces attacks like Sybil, sinkhole, selective forwarding, and Denial of Service (DoS), which threaten data transmission and network availability. Intrusion Detection Systems (IDSs) and trust-based routing protocols are among the proposed techniques in the literature to secure this layer. DLTs counter these threats through decentralized consensus and P2P communication models that eliminate reliance on centralized intermediaries, often a single point of failure in traditional systems. Trust and reputation mechanisms built on DLTs can detect and isolate malicious nodes, while the distributed nature of ledger updates ensures network robustness against routing-based manipulation and congestion.

The Application Layer is vulnerable to malicious code injection, unauthorized access, and data breaches, which compromise end-user privacy and service availability. DLTs can mitigate these threats by enabling fine-grained, rule-based access control and distributed storage mechanisms. Through cryptographic access permissions and smart contracts, DLT-based systems can enforce privacy-preserving data sharing and prevent unauthorized interactions with application services.

Among DLT solutions, Holochain emerges as a particularly suitable framework for IoT environments due to its agent-centric architecture, scalability, and ability to operate effectively on resource-constrained devices. This study proposes a Holochain-based framework as a decentralized and resource-efficient alternative to traditional architectures, capable of mitigating common attacks across the IoT stack. Holochain’s lightweight architecture and ability to scale without compromising security or performance while maintaining low overhead make it a promising candidate for IoT applications. This research explores the potential of Holochain in providing decentralized solution that aims to improve data integrity, trust, and security across distributed IoT networks.

Through this paper, we aim to provide an in-depth review of Holochain’s capabilities, compare it with other leading DLTs, and propose a conceptual framework, *HoloSec*, for securing IoT distributed networks. We also present a practical scenario in healthcare application, demonstrating Holochain setup and implementation. Ultimately, we explore the challenges of Holochain application in decentralized IoT networks and outline future research directions aimed at enhancing its interoperability, scalability, security, and regulatory compliance in IoT environments.

The main contributions of this paper are summarized as follows:

- A comprehensive review of DLTs in the context of resource-constrained IoT environments, highlighting the motivation for adopting Holochain over traditional blockchain.
- Introduction of the *HoloSec* framework, a conceptual security solution that integrates Holochain’s agent-centric architecture with advanced cryptographic mechanisms and machine learning for enhanced IoT network protection.
- Implementation of a Holochain-based IoT network tailored to a healthcare scenario, providing practical insights into real-world deployment.
- Empirical performance comparison between Holochain and traditional blockchain, demonstrating Holochain’s superior latency (50 ms publish, 30 ms retrieve) and throughput (20 TPS vs. blockchain’s 10 TPS) in a single-node setup.
- Identification of key research challenges and future directions related to the integration of Holochain in IoT, including scalability, interoperability, security, and regulatory compliance.

The remainder of this paper is organized as follows. Section 2 reviews the literature, provides a comparison of Holochain with other DLTs, including blockchain, Tangle (IOTA), Hashgraph, and Sidechains, and discusses recent applications of IoT networks in Holochain-based solutions. Section 3 introduces the *HoloSec* framework, a proposed Holochain security framework for IoT distributed networks, leveraging Holochain’s agent-centric architecture, advanced cryptography, and machine learning. Section 4 presents an experimental evaluation of Holochain in a healthcare IoT network scenario. Section 5 examines key challenges associated with Holochain and outlines future research directions aimed at enhancing its interoperability, scalability, security, and regulatory compliance in IoT environments. Section 6 concludes with a summary of findings and outlines future research directions.

## 2. Literature Review

This section reviews the existing literature comparing Holochain with other DLTs and outlines recent applications of Holochain in various IoT domains.

### 2.1. Comparison of Holochain with Other DLTs in IoT Networks

DLTs have revolutionized decentralized systems. Each DLT has unique characteristics that make it suitable for specific use cases. For IoT applications, factors like scalability, energy efficiency, latency, and decentralization play critical roles in determining the suitability of a particular DLT. Table 1 compares Holochain with other prominent DLTs: blockchain, Tangle (IOTA), Hashgraph, and Sidechains.

Holochain, with its agent-centric design, provides a scalable and decentralized alternative to traditional DLTs like blockchain, Tangle (IOTA), and Hashgraph. Unlike these systems, Holochain does not require global consensus, eliminating the energy-intensive processes of Proof of Work (PoW) or Proof of Stake (PoS). This makes it ideal for serverless IoT applications with millions of lightweight, autonomous devices. Each peer has its own ledger (local chain) and operates independently while interacting with others. Users control their identity and data, and encrypted P2P networks facilitate communication. Holochain’s lack of global consensus results in low latency and high throughput, addressing scalability and speed issues present in other DLTs like blockchain.

However, Holochain’s agent-centric model also introduces certain challenges, particularly in terms of interoperability and adoption. One significant limitation is the lack of established standards for Holochain, which complicates its integration with enterprise-grade systems. The authors in [21] systematically compare Ethereum and Hyperledger Fabric, emphasizing their use in attribute-based access control within IoT environments. The study highlights that both platforms benefit from robust ecosystems and established standards, like Ethereum’s ERC tokens and Hyperledger Fabric’s modular architecture, which provide versatility for various blockchain applications. Holochain lacks similar widely accepted protocols, which poses challenges for developers and enterprises looking for interoperability and consistency across platforms.

Since it diverges from traditional blockchain paradigms, integrating Holochain with existing systems, especially those reliant on global consensus mechanisms, presents significant complexities and costs. Holochain’s agent-centric approach offers promising solutions for decentralized data ownership but raises concerns about data consistency and security in adversarial environments due to the absence of a common ledger. As the authors in [22] discussed, in IoT networks, the lack of a unified ledger can be exploited by adversarial agents to create conflicting data states, increasing the risk of inconsistencies and undermining trust. They highlight that without a centralized mechanism to ensure coherence, managing distributed agents in hostile conditions requires robust trust management and validation strategies to mitigate risks effectively. This underscores the need for enhanced frameworks to maintain data integrity in decentralized systems like Holochain.

These concerns are compounded by the novelty of the Holochain framework, which is still in the process of gaining widespread adoption and developer support. The relatively small community and lack of established standards make it harder to build trust among enterprises and developers, hindering its scalability in large-scale IoT deployments compared to more established DLTs like IOTA, which has been specifically designed with IoT in mind. IOTA’s Tangle framework has already been successfully employed in real-world IoT applications, such as Bosch’s IoT devices and the +CityxChange smart city initiative, where it facilitates secure P2P energy transactions without intermediaries [23]. This demonstrates IOTA’s readiness for practical use, with a focus on scalability, energy efficiency, and data integrity. Holochain, while promising in terms of decentralized data ownership, remains less proven in such large-scale applications, especially in scenarios requiring high levels of consistency and security across distributed systems.

### 2.2. Applications of Holochain in IoT Networks

**Figure 2:**
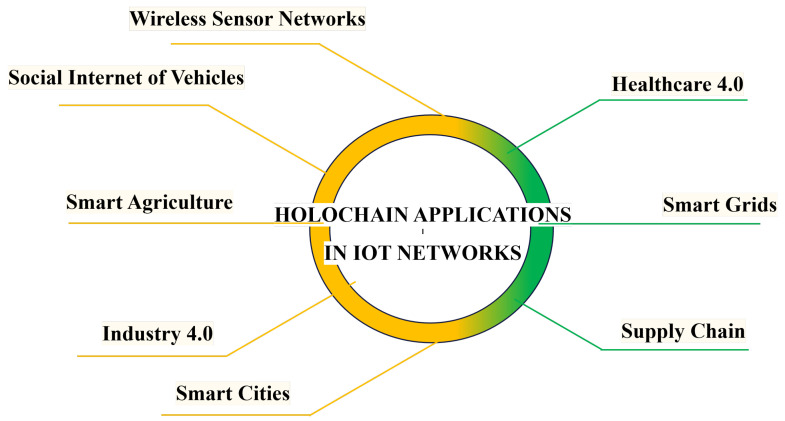
Holochain applications in IoT networks.

Decentralized architectures like Holochain have shown potential for improving security, privacy, and trust in various IoT networks. The agent-centric approach of Holochain allows each node to maintain control over its data, enhancing both security and privacy without the large storage requirements and data exchange constraints inherent in blockchain technology [24]. This section explores specific applications of Holochain in various IoT networks. Figure 2 illustrates IoT applications highlighted in green that have begun leveraging Holochain, while those in yellow, despite ongoing discussions, have yet to see recent practical implementations in the literature.

### 2.3. Supply Chain Management (SCM)

SCM primarily faces persistent issues such as lack of transparency, fragmented data ecosystems, and the dynamic nature of global markets [25,26]. Supply chains (SCs) often encounter critical performance bottlenecks that elevate costs, compromise product quality, and reduce operational efficiency [27,28]. Notable inhibitors include the bullwhip effect, where small demand fluctuations amplify up the Supply Chain (SC), influencing the launching of new product lines without agile coordination, overstocking or excessive inventory, and restrictive or siloed data flows.

These challenges can lead to inefficiencies where manufacturers are compelled to overproduce or overstock raw materials, increasing operational strain. Furthermore, limited interoperability and delayed data sharing in complex global SC networks hinder real-time visibility and slow down the seamless movement of goods and services.

DLTs have shown promising potential in alleviating these bottlenecks. DLT enables greater transparency, immutability, and decentralization, which are pivotal in enhancing the resilience and reliability of SCM systems [29]. For instance, in IoT-enabled SCs, the integration of Holochain offers improvements in data integrity, provenance, and the authenticity of goods. Its agent-centric model allows each participant to independently validate and verify product-related data, maintaining tamper-proof and trustworthy records across the network. A practical example is demonstrated in [30], where Holochain ensures end-to-end traceability in food SCs, from farm to consumer, enhancing food safety, compliance, and consumer trust.

Holochain provides a significant advantage for SCM by eliminating the requirement for global consensus, which in turn enables horizontal scalability through independently operating nodes. Its agent-centric architecture empowers each participant to maintain fine-grained control over their data, allowing them to selectively share information.This ensures confidentiality and supports regulatory compliance, particularly important for handling sensitive information such as pricing, supplier agreements, or customer data. Unlike traditional blockchain systems, Holochain does not rely on mining, tokens, or energy-intensive consensus mechanisms, making it lightweight, energy-efficient, and faster. These characteristics translate to lower operational costs, reduced environmental impact, and quicker transaction processing. Holochain’s architecture is resilient to localized failures, as nodes operate with eventual consistency and the system is self-healing and context-aware, key benefits for managing complex, geographically dispersed, and fragmented SCs.

### 2.4. Smart Energy and Decentralized Power Grids

Holochain can address the issue of data confidentiality and security in P2P energy exchange networks by enabling safe, decentralized transactions between energy producers and consumers. According to [31], Holochain may increase network resilience to outages in decentralized electrical networks by distributing control among network participants, addressing unique failure points, and ensuring network continuity in intelligent networks. One essential component of the decentralized Microgrid Transactive Energy Systems (MG-TESs) is DLT. It involves an information system that uses protocols to access, validate, update, and store records in a transparent and secure manner through a P2P decentralized computer network that may be spread across multiple locations [32]. The use of distributed data storage and management structures, such as DLT, in the energy sector has garnered a lot of attention recently [33]. This opens up new possibilities, such as managing micro-resources, aggregating distributed resources, enabling trade restrictions, integrating electromobility, or implementing provenance strategies [32]. Holochain has been explored as a promising framework for the decentralized operation of internet-connected devices and objects. Unlike traditional blockchain systems, Holochain allows a subset of users to validate or share transactions, eliminating the need for consensus across the entire network and thus improving efficiency and scalability [34].

Holochain can address data privacy and security in P2P energy trading networks by allowing secure, decentralized transactions between energy producers and consumers. For example, Ref. [35] suggested that Holochain can enhance fault tolerance in decentralized power grids by distributing control among grid participants, mitigating single points of failure, and ensuring operational continuity in smart grids.

### 2.5. Wireless Sensor Networks (WSNs)

WSNs typically consist of spatially distributed micro-devices, known as sensor nodes, which collaboratively monitor physical or environmental conditions [36]. However, these nodes are inherently constrained in terms of energy supply, processing power, memory, and communication range. These limitations make it challenging to implement robust data management and security mechanisms, leaving WSNs vulnerable to cyber-attacks and data integrity issues [37,38,39,40]. Unlike traditional blockchain architectures that rely on energy-intensive consensus mechanisms and centralized control, Integrating Holochain into WSNs employs a distributed hash chain model where each node maintains its own chain of validated data which aligns well with the limited resources of sensor nodes.

In a Holochain-enabled WSN, each sensor node can independently validate, store, and share data with its peers, promoting decentralized trust and eliminating the need for a central authority. This enhances data integrity and network resilience against common attack vectors such as data tampering, spoofing, and DoS attacks. Holochain’s architecture reduces the overhead typically associated with data synchronization and consensus, making it a scalable and energy-efficient framework for secure data exchange in resource-constrained sensor networks.

### 2.6. Social Internet of Vehicles (SIoV)

The SIoV integrates vehicular networks with the social behavior of users, enabling enhanced vehicle-to-vehicle (V2V), vehicle-to-infrastructure (V2I), and vehicle-to-everything (V2X) communication [41]. By combining vehicular communication technologies with the dynamics of social networking, the SIoV aims to transform the driving experience through improved road safety, traffic coordination, travel efficiency, and real-time infotainment services [42]. Despite the potential benefits, existing architectures primarily rely on centralized cloud and semi-centralized edge computing systems, which pose limitations in terms of latency, scalability, and security, particularly when managing the vast amount of data generated by connected vehicles [43].

Holochain can effectively overcome these challenges by enabling secure, P2P communication among vehicles without relying on a central server. For instance, it supports decentralized trust management by allowing each vehicle to maintain and validate its own interaction history and reputation, enabling autonomous trust assessments [44]. Malicious behaviors, such as false data injection or identity spoofing, are mitigated through peer validation and application-specific rules—invalid or harmful data is rejected before dissemination. Repeated misbehavior leads to exclusion from the network, not merely a reduced trust score.

Holochain supports real-time, privacy-preserving data exchange, allowing vehicles to share critical information like traffic congestion or hazardous road conditions without exposing sensitive user data. Its architecture enables secure, context-aware social services such as dynamic carpooling, collaborative trip planning, and decentralized incentives. In emergency scenarios, vehicles can form resilient ad hoc networks, ensuring continuity of communication.

### 2.7. Healthcare 4.0

The integration of IoT technologies into healthcare has ushered in the era of Health 4.0, where interconnected networks link patients, healthcare professionals, medical facilities, and suppliers to create a cohesive and intelligent healthcare ecosystem [45,46]. Despite these advancements, the sector faces key challenges such as patient dissatisfaction, escalating costs, medication traceability, and the imperative to safeguard sensitive medical data. The decentralized nature of healthcare systems further complicates the execution of processes in a manner that is reliable, secure, efficient, and transparent.

The convergence of DLTs and the IoT offers a promising solution by enabling secure tracking of medical products and protecting patient data through decentralized P2P networks. However, traditional blockchain implementations often introduce significant computational overhead, rendering them less suitable for resource-constrained IoT healthcare environments [47]. Holochain presents itself as a viable alternative for secure, decentralized, and scalable healthcare systems capable of managing sensitive data and complex, multi-stakeholder interactions. For instance, Ref. [48] introduced a Holochain-based IoT healthcare framework that effectively addresses data security and confidentiality while reducing complexity and resource demands. Their comparative analysis indicates that Holochain achieves security and data integrity levels comparable to blockchain-based solutions but with diminished computational overhead.

Further research [47] proposed a hybrid DLT model that integrates both blockchain and Holochain to enhance privacy and security within IoT healthcare ecosystems. This approach mitigates the individual limitations of each technology, such as recognition delays, memory consumption, and computational costs, while leveraging their respective strengths.

### 2.8. Smart Cities

Smart cities harness digital technologies to enhance urban living and streamline municipal services [49,50]. Central to this evolution is the IoT, which facilitates real-time data collection and analysis, enabling cities to monitor infrastructure, manage assets, and respond proactively to community needs [51]. However, challenges such as data security, transparency, and privacy persist. While blockchain has been explored to address these issues [52], its limitations in scalability and energy efficiency prompt the exploration of alternative solutions [49,52,53].

Holochain emerges as a compelling alternative, where its architecture supports various applications, including secure data sharing among city departments, transparent citizen engagement platforms, and efficient management of urban resources. Its resilience to inconsistent connectivity ensures reliable operation even in environments with variable network conditions. By enabling secure, transparent, and efficient data exchange, Holochain contributes to the development of sustainable and responsive smart city infrastructure, fostering greater trust and collaboration among stakeholders.

### 2.9. Agriculture

The integration of IoT technologies into agriculture has revolutionized farming practices by providing real-time data on crop health, soil moisture, and weather conditions, enabling farmers to make informed decisions and optimize operations [54,55]. However, traditional agricultural systems often face challenges such as data tampering, lack of transparency, and inefficiencies in record-keeping, which can lead to issues like theft, unavailability, and damage to records [31]. DLTs can significantly enhance agricultural product traceability from farm to market, ensuring all stakeholders have access to accurate, tamper-proof information while improving product safety and quality throughout the SC. For example, blockchain has been employed in [56] to develop a framework that continuously monitors crop, soil, and environmental conditions, supporting sustainable and eco-friendly farming practices. The system used blockchain’s inherent immutability and data provenance to ensure the authenticity and traceability of agricultural produce, while also enabling transparent decision-making and reliable food certification across the agri-chain. However, traditional blockchain implementations often encounter significant limitations in Food and Agriculture Infrastructure due to increased latency, high resource consumption, and elevated communication overhead, as discussed in [57]. These challenges hinder the system’s ability to meet large-scale, real-time identity authentication demands and contribute to substantial electricity consumption, ultimately affecting the scalability and practicality of blockchain deployment in real-world agricultural scenarios.

Recently, Holochain has emerged as a promising alternative in agriculture. For example, a Holochain-based, IoT-enabled agri-food traceability system was proposed for the dairy industry, demonstrating greater sustainability and efficiency compared to traditional blockchain models [30]. Beyond traceability, Holochain enables secure and private data sharing among SC stakeholders, including farmers, suppliers, certifying authorities, and consumers, ensuring the authenticity of health certificates and compliance with food safety regulations. Unlike blockchain platforms that rely on smart contracts, Holochain enforces data integrity and rules of interaction through custom validation logic and P2P protocols defined within its distributed applications. These mechanisms ensure that only certified products are admitted to the decentralized market, preventing the sale of food without proper documentation and establishing decentralized control based on verifiable credentials and cryptographic proof. It also enables the creation of decentralized marketplaces, allowing farmers to directly connect with buyers, reducing intermediaries and ensuring fair pricing. Holochain can also optimize the use of resources such as water, fertilizers, and pesticides through real-time monitoring and data analysis and protect land ownership records against unauthorized access and corruption by maintaining them on a secure, decentralized ledger.

### 2.10. Industrial IoT (IIoT) Networks

Advances in intelligent, machine-to-machine communication have enabled flexible manufacturing in Industry 4.0. However, increased interconnectivity exposes industrial systems to cyber threats such as remote sabotage, risking operational disruptions and financial losses. Ensuring execution integrity and system reliability is thus critical [58]. Although DLTs like blockchain have been considered to address these risks, they often face challenges with scalability, interoperability, data privacy, and environmental impact [14,59].

The decentralized nature of Holochain ensures that IIoT systems can operate effectively even in environments with intermittent connectivity. Devices can continue to function autonomously, updating the network when a connection becomes available, thereby reducing vulnerabilities associated with centralized systems. This resilience is particularly beneficial for applications like predictive maintenance, where uptime and real-time monitoring are essential. Holochain’s flexible and agent-centric architecture enables the development of interoperable applications that integrate with existing industrial systems, supporting a wide range of IIoT use cases such as remote monitoring, quality assurance, and SC optimization. Its scalable design efficiently handles the high volumes of real-time data generated within Industry 4.0 environments, while its lightweight, energy-efficient framework ensures low operational costs, making it a sustainable and cost-effective solution for modern industrial applications.

## 3. *HoloSec*: Proposed Conceptual Holochain Security Framework for IoT Distributed Networks

**Figure 3:**
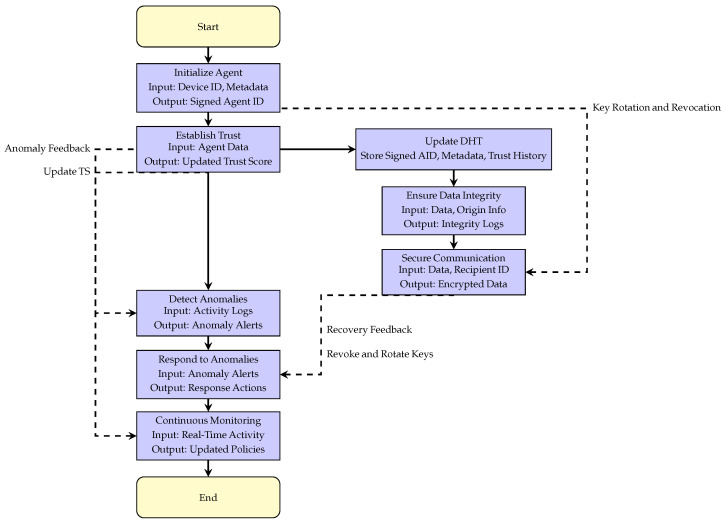
Block diagram of HoloSec framework.

The *HoloSec* framework offers a robust security solution for IoT networks, leveraging Holochain’s agent-centric architecture, advanced cryptography, and machine learning (ML). This decentralized design reduces computational overhead and energy consumption while enhancing scalability, privacy, and security. Each device (also called agent) operates autonomously, maintaining its own local ledger, ensuring data integrity and immutability without centralized storage. *HoloSec* supports having a mechanism for dynamic trust evaluation to assess agents behavior, adapting security measures as needed, while real-time anomaly detection powered by ML identifies and mitigates threats. The framework supports self-healing capabilities and seamless updates, ensuring ongoing security in evolving IoT environments. The proposed *HoloSec* framework provides an adaptive approach for securing IoT devices in a distributed environment by leveraging Holochain, ML, and cryptographic techniques. This framework enhances device trustworthiness, ensures data integrity, facilitates secure communications, and detects anomalies for proactive responses. Figure 3 depicts the block diagram of the *HoloSec* framework. The following outlines the key functionalities and processes involved.

### 3.1. Agent Initialization and Trust Establishment

The framework begins with the initialization of an agent (or IoT device), where a pair of public/private keys are generated using elliptic curve cryptography (ECC) for efficient and secure key management by using lightweight cryptographic libraries such as TinyCrypt or MicroECC. Lightweight cryptographic libraries such as TinyCrypt or MicroECC can be used to generate ECC key pairs directly on the IoT device. The agent’s identity (AID) is created by combining the device’s unique identifier (e.g., MAC address) with its public key, ensuring cryptographic uniqueness. This AID is then signed using the agent’s private key, creating a cryptographically verifiable identity. The signed AID is stored on the global shared Holochain Distributed Hash Table (DHT), ensuring decentralization and immutability. A local monitoring module can also be deployed on the edge device as a lightweight agent process written in C, Rust, or Python, depending on the IoT platform, and can even publish its metrics to the Holochain DHT or local ML model for Trust Score (TS) updates and anomaly detection. It will continuously track its behavior—monitoring metrics like uptime, response times, and participation in the network.

Once initialized, the agent’s trust is established through a dynamic TS mechanism. The TS is initialized to a default value (e.g., 0.5 on a scale of 0 to 1) and updated in real-time based on the agent’s behavior using the local monitoring module, with the following examples.

- Positive behaviors (e.g., timely data sharing, valid transactions) increase the TS.
- Negative behaviors (e.g., delayed responses, invalid data) decrease the TS.

If the TS falls below a predefined threshold (e.g., 0.3), the agent is flagged as untrusted, and adaptive security protocols are applied, such as isolating the agent or requiring additional authentication.

### 3.2. Data Integrity Verification

Data integrity is ensured through a combination of cryptographic hashing and Chain of Custody (CoC) records. When an agent collects or transmits data, a cryptographic hash (e.g., SHA-256 or BLAKE2) of the data is generated, creating a unique fingerprint. A CoC record is then created, containing:

- The data hash.
- Metadata (e.g., timestamp, origin ID).
- Signatures from a select group of trusted peer agents based on their TS.

The CoC record is signed by the originating agent and a trust-based subset (i.e., at least two trusted peers). Having a subset of nodes validate transactions and co-sign records is the core idea of Holochain, which significantly reduces computational and energy costs [60]. The signed record is then stored on the global shared DHT, ensuring decentralization and immutability.

To verify data integrity, the recipient agent:

- Retrieves the CoC record from the DHT.
- Validates the signatures using the public keys of the originating and peer agents.
- Recomputes the hash of the received data and compares it with the hash in the CoC record.

If discrepancies are detected (e.g., mismatched hashes), an alert is triggered via a gossip protocol, and predefined security protocols are activated, such as notifying stakeholders, isolating the compromised agent, and initiating data rollback or recovery.

### 3.3. Secure Communication and Authentication

Communication between agents is secured using asymmetric cryptography, such as RSA or ECC. Data is encrypted with the recipient’s public key and signed with the sender’s private key, ensuring confidentiality, authenticity, and non-repudiation, with the following examples.

- The sender encrypts the data using the recipient’s public key (e.g., RSA or ECC).
- The sender signs the encrypted data using its private key.
- The recipient decrypts the data using its private key and verifies the sender’s signature using the sender’s public key.

Mutual authentication is performed to ensure that both parties are legitimate. This involves the exchange of public keys between the sender and recipient, followed by verification of each party’s identity by validating the received public key against the Holochain global DHT.

To further enhance security, encryption keys can be periodically rotated using a key rotation protocol. Key rotation involves re-encrypting data using a newly generated key to replace the old one [61]. For instance, a new key pair can be generated every X hours. The new public key is then broadcast to all trusted peers and stored on the DHT, while the old private key is securely deleted to prevent unauthorized access.

### 3.4. Anomaly Detection and Response

Anomaly detection is powered by lightweight ML models deployed either on edge devices or via a centralized analytics node. These models are trained on historical agent behavior to distinguish between normal and suspicious activity. As real-time logs are analyzed, patterns such as unusual traffic, irregular timing, or protocol violations trigger immediate alerts.

Upon detecting anomalies, the system

- Logs the incident immutably on the global DHT to ensure transparency and traceability.
- Adjusts the agent’s TS in an adaptive manner, proportional to the severity of anomalous behavior.
- Initiates predefined security actions, including isolating affected agents, notifying stakeholders, and activating automated recovery workflows.
- Employs key rotation to revoke potentially compromised keys and replace them with new cryptographic pairs. This process not only re-establishes secure communication but also enforces access control by preventing further use of the invalidated credentials.

### 3.5. Continuous Monitoring and Adaptation

The framework continuously monitors agent behavior, system health, and environmental metrics using real-time monitoring tools integrated into each node or via centralized dashboards built with platforms like Grafana. Metrics such as transmission rates, connection stability, and TS fluctuations are visualized to provide administrators with actionable insights. Additionally, the framework integrates external threat intelligence feeds to update anomaly detection models with emerging threat patterns. Firmware updates and security patches are distributed via over-the-air (OTA) mechanisms like Mender or custom secure update services, using signed packages to ensure authenticity. As new data becomes available, TSs are dynamically updated, and policies are adapted, ensuring that security measures remain effective. The following are examples.

- Agents with consistently high TS values are granted additional privileges (e.g., access to sensitive data).
- Agents with low TS values are subjected to stricter security protocols (e.g., additional authentication steps).

## 4. Holochain Experiment for Healthcare IoT Network

**Figure 4:**
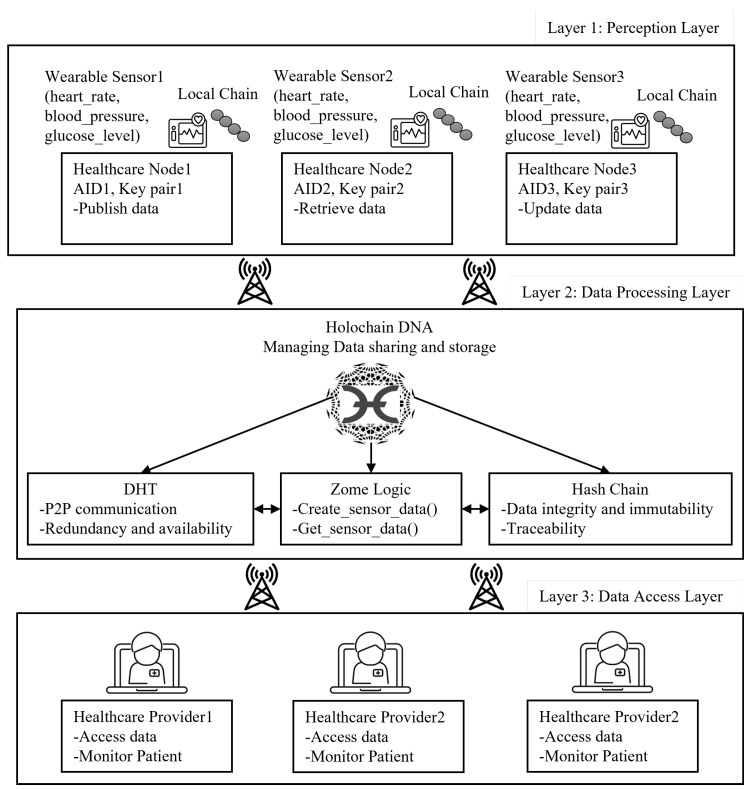
Holochain-based healthcare IoT system model.

This section describes the setup and implementation for a Holochain-based IoT network designed for a healthcare application scenario. The network integrates wearable sensors to monitor critical health parameters such as heart rate, blood pressure, and glucose levels, ensuring secure and real-time data sharing between healthcare providers and patients. The experiment demonstrates the potential of Holochain to decentralize the collection and sharing of health data. Figure 4 illustrates the system model for the Holochain-based healthcare scenario as a three-layer architecture: layer 1 represents the perception or data collection layer that consists of the wearable sensors act as data generators, continuously measuring physiological parameters and forwarding them to healthcare nodes. Layer 2 is the data processing layer that processes, stores, and manages data securely. DHT ensures P2P data storage, retrieval, and security through cryptographic hash chains, while Zome logic governs data management, defining functions such as create_sensor_data for publishing health data and get_sensor_data for retrieving health data. Layer 3 is the data access layer, where healthcare providers (hospitals, doctors, caregivers) access patient data securely from the decentralized Holochain network for monitoring and decision-making.

### 4.1. Experimental Setup and Configuration

The experiment was carried out by creating a custom DNA to manage the sensor data (Listing 1). The development environment was prepared using the Holochain Launcher and CLI tools, with Rust as the primary language. A local test network was deployed with three simulated healthcare agent nodes connected to the same DNA instance. Each agent node was assigned a unique AID and key pair. Secure messaging and authenticated data operations were performed using these credentials.

A new DNA project was initialized as follows:


**Listing 1.** DNA Structure.

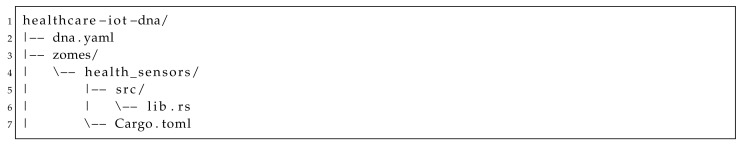




Zome logic was implemented to allow the capture and sharing of healthcare data such as heart rate, blood pressure, and glucose levels. Listing 2 represents the Rust code that defines the Zome logic for handling sensor inputs:


**Listing 2.** Zome Logic for Healthcare IoT Network.

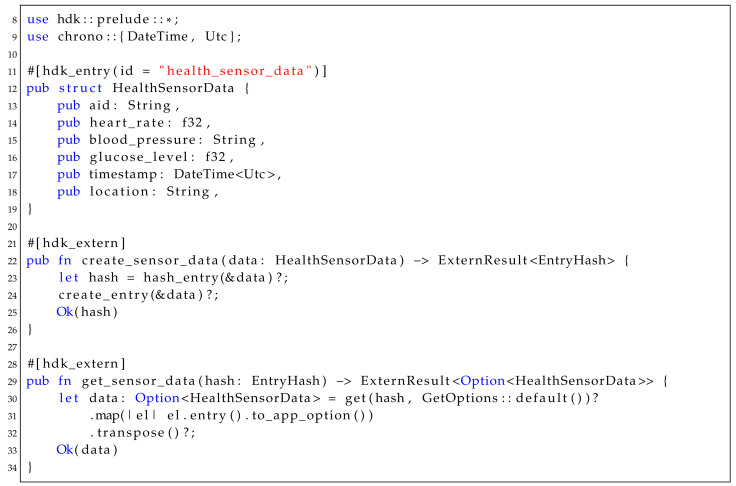




This logic enables each agent to publish and retrieve health data entries. The following steps demonstrate how the nodes interact:

- Node 1: Monitored heart rate and blood pressure during a patient check-up. Data was published using the command:

    holochain-cli call --fn=create_sensor_data --args='{
      "aid": "W-sensor-1",
      "heart_rate": 75.0,
      "blood_pressure": "120/80",
      "glucose_level": 95.0,
      "timestamp": "2025-04-16T14:23:00Z",
      "location": "Room 101, Clinic A"
      }' --id=node1
- Node 2: Retrieved the data shared by Node 1 to verify the patient’s condition. The hash of the entry created by Node 1 was used as input for the retrieval command:

    holochain-cli call
      --fn=get_sensor_data
      --args='{"hash": "<entry_hash>"}'
      --id=node2
- Node 3: Published glucose data to update the patient’s monitoring status. This node also verified the integrity of the data by retrieving and validating the timestamp and physiological parameters.

### 4.2. Results and Comparison

In this experiment, Node 1 published health data, Node 2 retrieved it, and Node 3 updated the data. We compared Holochain with blockchain in terms of validation latency and throughput. The blockchain experiment was conducted using Ethereum with Proof of Authority (PoA). PoA is preferred for private or permissioned blockchain networks, which are common in IoT and enterprise environments [62,63]. A smart contract was deployed to store and retrieve health data, and geth (Ethereum CLI) was used for interaction. Our preliminary results, presented in Table 2, show that Holochain achieves a latency of around 50 ms to publish data and 30 ms to retrieve it, with a throughput of around 20 TPS for a single node. In contrast, blockchain exhibits higher latency (around 200 ms to publish and 100 ms to retrieve) and lower throughput (around 10 TPS).

**Figure 5:**
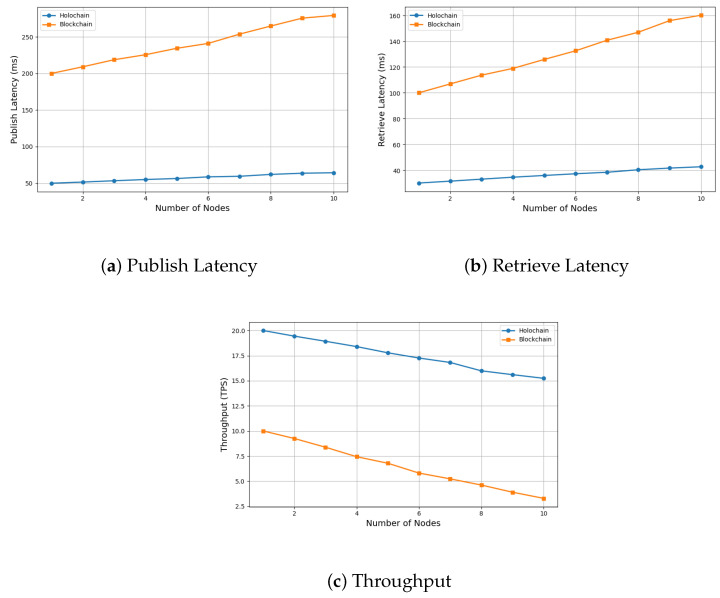
Simulation results for latency and throughput of Holochain vs. blockchain.

To evaluate scalability, we simulated an increasing number of nodes, from 3 up to 10 (Figure 5). The results show that Holochain maintains relatively stable performance as the network grows. At 10 nodes, Holochain sustains an average throughput of approximately 15 TPS, with only a modest increase in publish and retrieve latencies (up to 65 ms and 45 ms, respectively), while the blockchain system experiences significant performance degradation with increasing network size. By 10 nodes, its throughput drops to three TPS, and latencies increase substantially, publish latency exceeds 280 ms, and retrieve latency approaches 160 ms. These results highlight the scalability limitations of traditional blockchain platforms in latency-sensitive healthcare IoT applications.

## 5. Challenges and Open Research Directions

This section outlines the key challenges and open research directions when integrating Holochain into IoT networks. While Holochain offers significant advantages over traditional blockchain systems, such as scalability and agent-centric data management, several technical and regulatory hurdles must be addressed to realize its full potential in IoT applications.

### 5.1. Interoperability

Integrating Holochain with existing IoT systems and protocols presents a significant challenge. IoT networks often rely on standardized protocols such as MQTT, CoAP, and HTTP for interoperability. These protocols may not be directly compatible with Holochain’s unique decentralized architecture, which uses DHTs for data storage and retrieval. Holochain’s DHT-based P2P model requires translation layers for seamless integration. For example, middleware solutions could translate between Holochain’s agent-centric model and the request–response or publish–subscribe models of conventional IoT protocols. Such an approach would allow Holochain-based IoT systems to interact with legacy devices while preserving decentralization and data integrity. Future research in this area could focus on the following:

- Protocol adapters that translate MQTT/CoAP messages into a format that Holochain can process.
- Edge computing integration, where edge devices serve as intermediaries, preprocessing data before DHT storage.
- Decentralized identity and access management, ensuring secure device authentication within an agent-centric network.

### 5.2. Tooling Maturity and Ecosystem Development

Holochain’s ecosystem is still in a nascent stage and suffers from immature tooling and limited community support compared to more established DLTs like IOTA and Hyperledger. These mature platforms provide robust SDKs, middleware, testing tools, and extensive documentation, which ease development and deployment in IoT environments. Holochain’s relative lack of such resources slows development, increases onboarding difficulty, and complicates maintenance. Enhancing tooling, documentation, and community engagement is vital for wider adoption and competitiveness.

Future research directions include the following:

- Development of comprehensive SDKs, middleware, and standardized protocols to simplify integration with IoT systems and improve interoperability with other DLTs.
- Creation of advanced developer tools and resources, including debugging, testing, simulation environments, and detailed documentation.
- Fostering an active and supportive developer community by encouraging reusable components, plugin architectures, and feedback-driven iterative improvements in tooling and deployment practices.

### 5.3. Scalability in Large-Scale Networks

While Holochain offers scalability improvements over blockchain, challenges remain when scaling it across vast IoT networks, (e.g., smart cities with 10,000+ devices or large industrial applications) which might involve millions of devices generating terabytes of data daily. Future research directions include the following:

- Optimizing DHT performance to efficiently manage high-throughput environments and reduce lookup latency.
- Implementing adaptive sharding mechanisms to distribute workload dynamically and improve query efficiency, for instance, by dynamically partitioning the DHT by location or device type.
- Edge computing integration to process data locally, reducing latency and bandwidth consumption before storing data in the DHT.
- Developing load-balancing techniques to prevent hotspot nodes and ensure uniform data distribution across the network.
- Exploring hybrid approaches that combine Holochain with edge and cloud computing for efficient large-scale deployment.

### 5.4. Security Threats

Despite its decentralized nature, Holochain remains susceptible to various security threats [47]. One significant risk stems from the random selection of transaction approval authorities, which may inadvertently allow an attacker to be part of the network, thereby enabling adversarial control over critical verification processes. This vulnerability can lead to network poisoning, where malicious nodes manipulate data consistency, disrupt consensus, or facilitate fraudulent transactions. The agent-centric model presents risks such as Sybil attacks, where adversaries generate multiple identities to undermine trust mechanisms. The DHT, while enhancing scalability, introduces additional attack vectors, including data tampering, unauthorized access, and eclipse attacks that isolate nodes from accurate network state information. To address these security concerns, future research should focus on the following:

- Developing secure authority selection mechanisms for transaction approval, incorporating trust metrics, stake-based incentives, and decentralized reputation models to minimize adversarial influence.
- Implementing decentralized authentication through strong identity verification techniques to prevent identity spoofing and unauthorized access, leveraging cryptographic signatures and decentralized identity frameworks.
- Introducing trust-based or stake-weighted models to restrict the influence of malicious nodes and enhance network resilience.
- Utilizing advanced cryptographic methods such as zero-knowledge proofs and homomorphic encryption to ensure data privacy, integrity, and secure computation within the network.
- Employing real-time ML anomaly detection by training models on Holochain’s source chain history to identify suspicious patterns such as Sybil attacks, collusion, and data injection threats.

### 5.5. Data Management and Storage

Decentralized data management in Holochain is challenging in data-intensive IoT applications. Managing large volumes of data across distributed agents can lead to issues in data consistency, storage overhead, and retrieval speeds. For example, in the healthcare IoT, real-time access to patient data is critical, and delays or inconsistencies could have serious consequences. Future research directions for optimizing data management in Holochain-based IoT systems include the following:

- Developing hybrid architectures that combine DHTs with centralized caching and adaptive data replication strategies to improve data retrieval speeds and balance consistency and availability in decentralized environments.
- Investigating efficient indexing techniques and consensus-free synchronization mechanisms to enhance query performance and ensure data consistency in large-scale distributed networks and agent-centric architectures.
- Strengthening DHT integrity by implementing verifiable storage mechanisms, cryptographic commitments, and tamper-proof logging to ensure data authenticity and resist manipulation, including hierarchical and off-chain storage, time-series compression, and GDPR-compliant data deletion.

### 5.6. Regulatory and Privacy Considerations

The use of decentralized technologies like Holochain raises privacy and regulatory challenges, especially as IoT devices collect sensitive data. Ensuring compliance with privacy laws like GDPR and CCPA is essential. For example, GDPR’s right to be forgotten conflicts with the immutability of decentralized systems. Research should explore how Holochain can balance regulatory compliance with privacy and data integrity. Key research directions include:

- Developing mechanisms to support the right to be forgotten while preserving decentralization principles.
- Implementing privacy-preserving techniques such as zero-knowledge proofs, differential privacy, and secure multi-party computation.
- Designing regulatory-compliant data governance models for decentralized IoT ecosystems.
- Creating auditing and compliance monitoring frameworks tailored for decentralized architectures.
- Investigating policy-driven access control mechanisms to ensure legal and ethical data usage.

## 6. Conclusions

Holochain presents a promising solution for securing distributed IoT networks. Its decentralized, agent-centric approach enhances security and user control over data, making it well suited for a wide range of IoT applications. In this paper, we reviewed various DLTs, including blockchain, Tangle (IOTA), Hashgraph, and Sidechains, with a particular focus on Holochain. We also explored the applications of Holochain in IoT networks, covering use cases in supply chains, healthcare, and smart grids.

Additionally, we proposed *HoloSec*, a conceptual security framework for IoT networks that leverages Holochain’s agent-centric architecture, advanced cryptographic techniques, and ML. By combining Holochain’s decentralized nature with cryptographic protections and ML-based anomaly detection, HoloSec provides a highly resilient, autonomous, and scalable security framework, ensuring the reliability, data integrity, and trustworthiness of IoT networks.

Furthermore, we presented an experimental study on a Holochain-based IoT network designed for a healthcare application. We discussed the setup, implementation, and results, highlighting the potential benefits and challenges of adopting Holochain in real-world scenarios. Finally, we examined key challenges associated with Holochain and outlined future research directions to enhance its interoperability, scalability, security, and regulatory compliance in IoT environments.

## Figures and Tables

**Table 1:** Comparison of Holochain vs. other DLTs in IoT networks.

DLT Technology	Blockchain	Tangle (IOTA)	Hashgraph	Sidechains	Holochain
**Consensus Mechanism**	PoW, PoS, or Delegated PoS [8]	DAG where transactions validate each other [9]	Gossip protocol and Virtual Voting for BFT [10]	Independent consensus; varies by implementation (e.g., PoW, PoS) [11]	Agent-centric with local validation, no global consensus [12]
**Scalability**	Limited by block size and mining bandwidth	High; scales with more participants [9,13]	High; efficient communication	Depends on parent chain’s scalability	Very high; nodes operate independently [14]
**Latency**	High; delayed by mining/validation [15]	Low; transactions validate directly	Very low; near-instantaneous finality [16]	Variable; depends on implementation	Very low; local transactions are processed instantly
**Resource Efficiency**	High energy consumption (e.g., PoW) [8]	Low; no mining overhead	Low; avoids mining requirements	Dependent on parent chain	Very low; lightweight operations
**Security**	Vulnerable to 51% attacks [17]	Resilient; majority network control required	BFT	Independent security; separate trust model	Local integrity
**Decentralization**	High but centralized mining pools exist	High; designed for IoT	High; all nodes contribute equally	Variable; dependent on parent chain	High; agents independently own data
**Data Ownership**	Shared across nodes [8]	Shared ownership	Shared ownership	Inherited from parent chain	Agent-centric; users own their data
**Throughput**	Moderate (7–20 TPS for Bitcoin, 1000+ TPS for modern blockchains)	High; parallel processing of transactions	Very high; thousands of TPS	Variable; depends on design	Very high; no bottlenecks from global consensus
**IoT Suitability**	Limited by scalability and latency	Excellent; tailored for lightweight IoT devices	Suitable but resource-intensive	Moderate; specific to use case	Excellent; lightweight, agent-centric design
**Key Challenges**	Scalability, energy consumption	Immaturity; orphaned transactions	Complexity; proprietary licensing	Interoperability with main chain	Adoption and interoperability
**Platforms/Languages**	Bitcoin, Ethereum, C++, Go, Solidity	IOTA Rust, Go, C [18]	Hedera Java, JS	Polkadot, Cosmos Rust, Go [19]	Holo Rust [20]

**Table 2:** Holochain vs. blockchain comparison summary.

Performance Metric	Holochain	Blockchain
Latency	50 ms (publish), 30 ms (retrieve)	200 ms (publish), 100 ms (retrieve)
Throughput	20 TPS (stable)	10 TPS (decrease with more nodes)
Network Overhead	Low (P2P/DHT)	High (Global broadcast)
Storage Growth	Linear per agent (Local + shared DHT)	Exponential (full ledger replication)
Consensus Mechanism	Agent-centric validation	Global PoA consensus
10-Node Test	15 TPS (+20% latency)	<5 TPS (2× latency)

## Data Availability

No new data were created or analyzed in this study.
